# Prospects for low-toxicity lead-free perovskite solar cells

**DOI:** 10.1038/s41467-019-08918-3

**Published:** 2019-02-27

**Authors:** Weijun Ke, Mercouri G. Kanatzidis

**Affiliations:** Department of Chemistry, Northwestern University, Evanston, IL 60208 USA

## Abstract

Since the 2012 breakthroughs^1–3^, it is now very much accepted that halide perovskite solar cells may have a strong practical impact in next-generation solar cells. The most efficient solar cells are using Pb-based halide perovskites. The presence of Pb in these devices, however, has caused some concerns due to the high perceived toxicity of Pb, which may slow down or even hinder the pace of commercialization. Therefore, the science community has been searching for lower-toxicity perovskite-type materials as a back-up strategy. The community is paying significant attention to Pb-free materials and has achieved promising results albeit not yet approaching the spectacular performance of APbI_3_ materials. In this comment, we summarize the present status and future prospects for Pb-free perovskite materials and their devices.

## Limitations of Pb-based halide perovskite materials

**Fig. 1:**
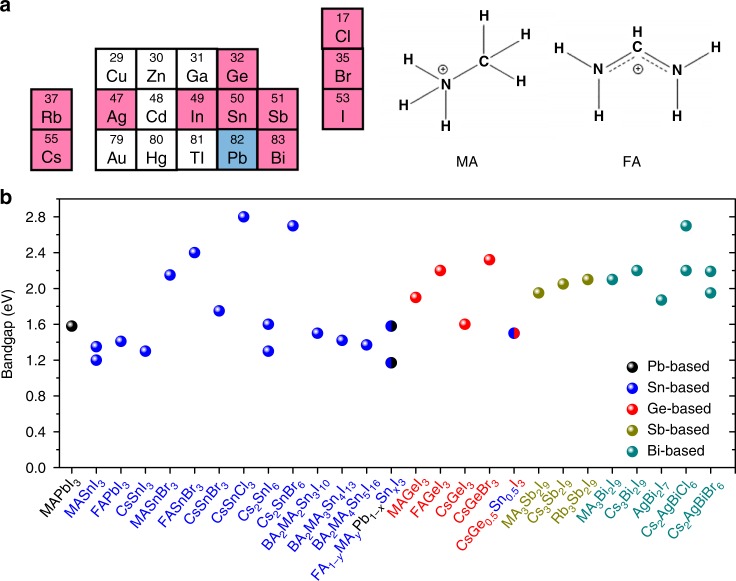
Potential materials as solar cell absorbers. **a** Potential A-site cations (organic MA and FA or inorganic Cs and Rb), metals, and halides (I, Br, Cl) for perovskite structure. **b** Bandgaps of various materials^4,6,8,9,18,19^. The suitable materials for solar cells should have direct bandgaps of around 1.1 to 2.0 eV

The optical and electrical properties of Pb-based perovskites look almost perfect for solar cells^4^. The latest efficiency of perovskite solar cells reached 23.7%^5^, outperforming that of Cu(In,Ga)(Se,S)_2_, CdTe, and Si-based solar cells. However, Pb-based perovskite solar cells have two main concerns: poor stability and high toxicity^6^. The stability challenge in these perovskites is being addressed through the use of two-dimensional (2D) perovskites as well as improved device engineering and encapsulation^7,8^. The development of low-toxicity Pb-free materials is of course always preferred in the solar cell market if performance is not too compromised. Ideal Pb-free candidates as solar cell absorbers should have low toxicity, narrow direct bandgaps, high optical-absorption coefficients, high mobilities, low exciton-binding energies, long charge-carrier lifetimes, and good stability. Fortunately, there are some low-toxicity constituents with perovskite structure having attractive properties, such as Sn/Ge-based halides, some double perovskites, and some Bi/Sb-based halides with perovskite-like structure, see Fig. 1^6,9^.

## Sn-based halide perovskite materials

Among these candidates, Sn-based perovskites have attracted the most attention due to their very similar properties, and the most promising performance achieved by their devices^10^. The representative methylammonium tin iodide (MASnI_3_), formamidinium tin iodide (FASnI_3_), and cesium tin iodide (CsSnI_3_) have direct bandgaps of around 1.20, 1.41, and 1.3 eV^11^, respectively, which are even narrower and more attractive than those of their Pb-based analogs. After exposure to ambient air, the Sn-based perovskites degrade to Sn^4+^ becoming SnO_2_, which is an environment-friendly material. A quick inspection of all the fundamental physical properties of the Sn-based perovskites and a comparison with those of the Pb-based ones reveal a remarkable similarity^11^. Therefore, at least in-principle these materials should be able to match the efficiencies of the APbI_3_ systems.

**Fig. 2:**
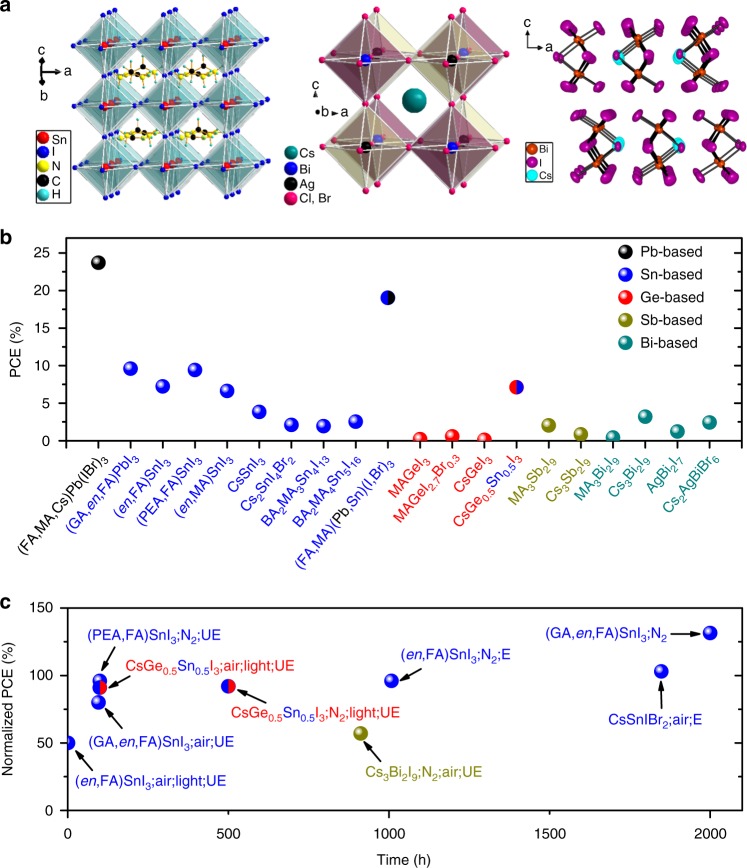
Comparison of structure, efficiency, and stability of different materials and devices. **a** Crystals structures of FASnI_3_ (left), Cs_2_AgBi(ClBr)_6_ (middle), and Cs_3_Bi_2_I_9_ (right)^19^. **b** Record efficiencies of representative solar cells using Pb, Sn, Ge, Sb, and Bi-based absorbers^6,9,13,18^. **c** Stability of representative Sn, Ge, and Bi-based perovskite solar cells under different conditions, i.e., encapsulated (E), unencapsulated (UE), ambient air, glovebox (N_2_), and light soaking^6,9,13,18^

As the first report on Sn-based perovskite solar cells which achieved power conversion efficiency (PCE) of around 6%^10,12^, many reports have focused on Sn-based perovskite solar cells^6^. So far, the highest efficiency of Sn-based perovskite solar cells has been reported to have reached 9.6% (Fig. 2)^13^. Taking a closer look at the photovoltaic parameters, Sn-based perovskite solar cells usually have high short-circuit current densities (*J*_sc_’s) of 20 to 25 mA cm^−2^ because of their low bandgaps. However, the average of open-circuit voltage (*V*_oc_) of the solar cells is only around 0.5 V, which is much lower than 1.1 V of the Pb-based analogs^6^. One of the main reasons for the low *V*_oc_ of the Sn-based perovskite solar cells should be the heavy p-type doping of the materials, attributed to the very facile and undesirable oxidization of Sn^2+^ to Sn^4+^, which acts as a p-type dopant in the structure resulting in too high dark-carrier concentration and very high photocarrier recombination.

Recently, it was shown that adding ethylenediammonium (*en*) cations give rise to Sn-based perovskites with new types of three-dimensional (3D) so-called hollow perovskites^14,15^, markedly tuning the bandgap from 1.3 to 1.9 eV. As a result, the 3D hollow Sn-based perovskite solar cells with 10% *en* addition achieved a much higher *V*_oc_’s and PCEs than the standard ASnI_3_ perovskites (0.48 V and 7.14% vs. 0.15 V and 1.4%)^14^. The record efficiency of 9.6% in a recent report was achieved by also adding 1% *en* combining 20% guanidinium (GA)^13^. Other cations, such as phenylethylammonium (PEA) and butylammonium (BA), resulting in low-dimensional structures are also attractive^8^. The representatives of 2D (PEA)_2_(MA)_*n*−1_Sn_*n*_I_3*n*+1_ and (BA)_2_(MA)_*n*−1_Sn_*n*_I_3*n*+1_ perovskites have much better stability because of the protection of the organic spacing layers. Especially, recent reports describe high performance usually using the 2D Sn-based perovskites with an inverted device structure^6^, giving the highest PCE of 9.4%. The use of both 3D hollow and 2D perovskite in the same films can significantly improve the stability of the Sn-based perovskite solar cells (Fig. 2c).

Cs_2_SnX_6_ (X = I, Br) is the most stable of Sn-based perovskites. This is a peculiar compound that has a stable Sn^4+^ and therefore does not oxidize, but it does not have a fully 3D perovskite structure, which is a major disadvantage. Cs_2_SnI_6_ has a narrow direct bandgap of 1.3 to 1.6 eV (Fig. 1)^16^. However, it has an isolated octahedron and have too low mobility and too high minority carrier-effective masses. If future progress is made in extending the carrier-diffusion lengths of this material, such as making high-quality films with big grains and less defects, it would be possible to increase the PCEs.

## Ge-based halide perovskite materials

There Ge-based perovskites also have similar properties with the Pb-based perovskites though not as similar as the Sn analogs^17^. In the AGeI_3_ perovskite family, CsGeI_3_ has the narrowest bandgap of around 1.6 eV (Fig. 1), whereas MAGeI_3_ and FAGeI_3_ have wider direct bandgaps of around 1.9 and 2.2 eV, respectively. Based on bandgap, it looks like only CsGeI_3_ is suitable as solar cell absorber. However, the current results in solar cell efficiency show that the Ge-based perovskites are even more inferior than the Sn-based perovskites. Making stable and high-performance solar cells using the Ge-based perovskites has been challenging.

More promising are Ge and Sn alloys, which have narrower bandgaps and better stability than the neat Ge-based perovskites^18^. All-inorganic high-quality CsSn_0.5_Ge_0.5_I_3_ perovskite films with a bandgap of 1.5 eV can be fabricated by thermal evaporation^18^, and solar cells gave a remarkable PCE of 7.11% (Fig. 2b). More remarkably, the CsSn_0.5_Ge_0.5_I_3_ solar cell without encapsulation can maintain 92% of its initial efficiency after 500 h of continuous operation under 1-sun illumination at 45 °C in N_2_ atmosphere (Fig. 2c). Even when exposed in ambient air, the solar cells can still retain 91% of its initial efficiency after 100 h of continuous light soaking. The improved stability was attributed to the protection of an ultra-thin GeO_2_ surface layer on the CsSn_0.5_Ge_0.5_I_3_ film.

## Other Pb-free halide perovskite materials

Apart from the directly analogous Sn and Ge-based perovskites, double perovskites with a formula of A_2_M^+^M^3+^X_6_ have been pursued (Fig. 2a)^9^. These 3D materials exhibit wider bandgaps of around 2 eV and tend to be more stable in air, however, they exhibit parity-forbidden transitions, indirect bandgaps, 0D electronic dimensionality, and large hole/electron-effective masses, leading to low mobilities and poor carrier transport. Therefore, solar cells based on Bi-based double perovskites have not achieved high *J*_sc_’s and PCEs, and fall in a similar category with the Cs_2_SnI_6_-based solar cells.

Other Bi and Sb-based compounds, e.g., Cs_3_Sb_2_I_9_ and Cs_3_Bi_2_I_9_, have similar bandgaps of around 2 eV^19^. These materials are stable in air, however, they do not have a 3D structure (Fig. 2a) or direct bandgaps. They also have strongly bound excitons, which inhibit free carrier generation. Therefore, they have similarly low mobilities, poor charge transport, and high resistivities of 10^10^ to 10^12^ Ω cm^19^. Solar cells using them as absorbers definitely have the same problem, which is very low *J*_sc_’s and PCEs. It looks that both these two types of Bi-based perovskite materials are not ideal as solar cell absorbers.

## Future prospects of Pb-free halide perovskite materials

On the basis of the above discussion, it looks like that only the Sn-based perovskites are promising for achieving high performance in the near future (Fig. 2b). However, the Sn-based perovskites have significantly worse stability than the Pb-based ones even with encapsulation. The Sn and Pb-based perovskites can borrow technologies from each other to further address the stability issues. We cannot yet judge, however, if they are viable candidates before the stability concern is addressed. Nevertheless, PCEs of 10% or higher are likely on the horizon. Once the Sn^4+^ oxidation issue is fully addressed and photocarrier recombination rates are suppressed to the levels of the APbI_3_ materials, *V*_oc_ of about 0.8 to 1.00 V should be achievable, which in turn will open the path to PCE beyond 15% and dramatically improve their future prospects to a viable lead-free contender. A neighbor of Sn-based perovskites, Ge-based perovskites, is not an ideal choice. However, the mixed Ge/Sn-based perovskites appear to be promising if future work can get the efficiencies higher than 10%. More work in needed to understand the ʽmagicʼ improvement of stability in future studies. One potential problem of Ge-based solar cells is the extremely high cost of Ge. So far, the Pb-free perovskite materials offer is poor dichotomy, (i) path to high-efficiency but poor stability (Sn^2+^-based), or (ii) good stability but low-performance (Sn^4+^/Sb/Bi-based etc.). Can we find an ideal Pb-free material with combined good optical and electrical properties with good stability? We hope more researchers will focus on this challenge. Although, the total Pb-weight of a 500 nm-thick perovskite film in solar cells may be small and the device engineering efforts will provide a safe and convincing solution including strategies for recycling Pb-based perovskites, the pursuit of Pb-free devices is certainly a worthy effort. The candidates should be not limited to halide perovskite families, new materials such as chalcogenide perovskites and non-perovskites with new structures are also worth investigating. Clearly, the new candidates should not only have ability to achieve high efficiencies but also be low-cost, scalable fabrication, and most importantly stable enough to reach the industrial standards, at least keeping initial high efficiencies after long-term operational lifetime (around 1000 h) under 85 °C and 85% relative humidity. The technologies of perovskite solar cells are young and exciting and their wheel of development rolls on; future progress will be bright especially as more research and investment efforts are undertaken.
